# In Silico and In Vitro Analysis of Multifunctionality of Animal Food-Derived Peptides

**DOI:** 10.3390/foods9080991

**Published:** 2020-07-24

**Authors:** Lourdes Amigo, Daniel Martínez-Maqueda, Blanca Hernández-Ledesma

**Affiliations:** 1 Departamento de Bioactividad y Análisis de Alimentos, Instituto de Investigación en Ciencias de la Alimentación (CIAL, CSIC-UAM, CEI-UAM+CSIC), Nicolás Cabrera 9, 28049 Madrid, Spain; lourdes.amigo@csic.es; 2 Departamento de Investigación Agroalimentaria, Instituto Madrileño de Investigación y Desarrollo Rural, Agrario y Alimentario (IMIDRA), 28800 Madrid, Spain; daniel.martinez.maqueda@madrid.org

**Keywords:** bioactive peptides, animal protein, multifunctionality, antioxidant activity, in silico, cell models

## Abstract

Currently, the associations between oxidative stress, inflammation, hypertension, and metabolic disturbances and non-communicable diseases are very well known. Since these risk factors show a preventable character, the searching of food peptides acting against them has become a promising strategy for the design and development of new multifunctional foods or nutraceuticals. In the present study, an integrated approach combining an in silico study and in vitro assays was used to confirm the multifunctionality of milk and meat protein-derived peptides that were similar to or shared amino acids with previously described opioid peptides. By the in silico analysis, 15 of the 27 assayed peptides were found to exert two or more activities, with Angiotensin-converting enzyme (ACE) inhibitory, antioxidant, and opioid being the most commonly found. The in vitro study confirmed ACE-inhibitory and antioxidant activities in 15 and 26 of the 27 synthetic peptides, respectively. Four fragments, RYLGYLE, YLGYLE, YFYPEL, and YPWT, also demonstrated the ability to protect Caco-2 and macrophages RAW264.7 cells from the oxidative damage caused by chemicals. The multifunctionality of these peptides makes them promising agents against oxidative stress-associated diseases.

## 1. Introduction

Non-communicable diseases (NCDs) such as cardiovascular and neurodegenerative disorders, cancer, and diabetes, are the principal cause of death and disability worldwide [1]. Most of these diseases are caused by environmental factors, with the diet being one of the main contributing factors. While the consumption of highly processed foods and sugar-sweetened beverages has been associated with a higher risk of these disorders, a healthy diet including functional foods may help in reducing or even preventing several NCDs [1,2]. Thus, in recent years, the search for bioactive food compounds and their use as substitutes of pharmacological treatments has intensified. Due to their desirable impacts on human health and limited side effects, bioactive peptides have become one of the most studied food components, being usually included into functional foods and nutraceuticals [3]. Once liberated from the source protein by enzymatic hydrolysis, gastrointestinal digestion, or food processing, bioactive peptides may act on different body systems exerting different functionalities such as antihypertensive, antioxidant, opioid, antithrombotic, hypocholesterolemic, anticancer, immunomodulatory, and antimicrobial activities, among others [4]. Moreover, it has been demonstrated that some food peptides are able to exert two or more bioactivities, acting on several systems at the same time [5]. This has made it so that multifunctional peptides have been recently recognized as more useful than peptides with single activity as they influence multiple cell processes, affecting different signaling pathways simultaneously [6]. Among food sources of multifunctional peptides, milk and meat proteins are considered some of the most studied [7]. As examples, caseinophosphopeptides have been reported to exert anticariogenic, antihypertensive, immune-enhancing, antigenotoxic, and cytomodulatory effects [3]. Whey protein lactoferrin and its derived peptide, lactoferricin, are well known by their anticancer, antitumor, immunomodulatory, and antimicrobial activities [8].

The rising evidence suggests a possible common pathophysiology among NCDs, with oxidative stress and hypertension as the main contributing factors [9,10]. Oxidative stress occurs when reactive oxygen species (ROS) overload the body’s defenses or when these defenses lose their capacity to react, leading to damage of essential cell components [11]. Experimental, clinical, and epidemiological studies have revealed that this status is involved in the development of NCDs such as arteriosclerosis, obesity, type 2 diabetes, inflammatory bowel disease, arthritis, neurological, liver, and renal disorders, and cancer [12]. Hypertension, defined as high blood pressure, is currently considered one of the major preventable risk factors linked to cardiovascular diseases [13].

The classical or empirical approach, also referred to as the in vitro method, is the most employed approach in peptide bioactivity screening. However, it requires exhaustive sample preparation and does not always allow the explicit identification of particular bioactive peptides. To overcome these major disadvantages, bioinformatics-driven (in silico) approaches have recently been developed. These strategies enable estimating potential precursor proteins through the calculation of quantitative descriptors, constructing profiles of the potential biological activity of peptide sequences, and predicting peptidic bonds susceptible to enzymatic hydrolysis [14]. Thus, in silico analyses have been recognized as playing a significant role in the process of bioactive peptides generation and identification [15]. However, there are many limitations associated with bioinformatic data, such as the extent, quality, and reliability of published information within databases. Moreover, the validation of attributed bioactive sequences, as well as the study of other aspects such as peptides’ stability, bioavailability, and mechanisms of action should be carried out to complete the provided information by in silico analyses. Therefore, different milk and meat-derived peptides that are similar to or share amino acids with previously described opioid fragments were selected and subjected to an integrated approach combining an in silico study and in vitro assays to evaluate their multifunctionality and mechanisms of action as basis of their future use in functional foods.

## 2. Materials and Methods

### 2.1. Materials

Triisopropyl silane, angiotensin-converting enzyme (ACE), fluorescein (FL), 2,2′-azinobis(3-ethylbenzothiazoline-6-sulfonic acid) diammonium salt (ABTS), 3-(4,5-dimethylthiazol-2-yl)-2,5-diphenyl tetrazolium bromide (MTT), dimethylsulfoxide (DMSO), dichlorofluorescin (DCFH), and Hank’s Balanced Salt Solution (HBSS) were purchased from Sigma-Aldrich (St. Louis, MO, USA). 2,20-azobis (2-methylpropionamide) dihydrochloride (AAPH) and 6-hydroxy-2,5,7,8-tetramethylchroman-2-carboxylic acid (Trolox) were obtained from Aldrich (Milwaukee, WI, USA). Abz-Gly-Phe (NO_2_)-Pro was purchased from Bachem Feinchemikalien (Bubendorf, Switzerland), and trifluoroacetic acid (TFA) was obtained from Scharlau (Barcelona, Spain). High-Glucose Dulbecco’s Modified Eagle Medium (DMEM), fetal bovine serum (FBS), and penicillin/streptomycin/amphotericin B solution were purchased from Biowest (Kansas City, MO, USA). A 1% non-essential amino acids solution was from Lonza Group Ltd. (Basilea, Switzerland).

The milk and meat-derived peptides used in this study (Table 1) were synthesized by the conventional Fmoc solid-phase synthesis method using an Applied Biosystems model 433A synthesizer (Foster City, CA, USA). The cleavage of the peptides from the polystyrene-based resin (Applied Biosystems, Foster City, CA, USA) was carried out with TFA, triisopropyl silane, and MilliQ water for 2 h. The analysis of peptides was carried out by high performance liquid chromatography coupled to tandem mass spectrometry (HPLC-MS/MS) analysis using an Agilent 1100 HPLC System (Agilent Technologies, Waldbron, Germany) connected online to an Esquire 3000 ion trap (Bruker Daltonik GmbH, Bremen, Germany) and equipped with an electrospray ionization source. The identification of peptides and their purity were determined through mass comparison and peak integration, respectively (Supplementary Figure S1). Finally, peptides were dissolved in 10% acetic acid, freeze-dried, and kept at −20 °C until further analysis.

### 2.2. Peptide Screening by In Silico Analysis

Synthetic peptides were subjected to in silico analysis by using the Milk Bioactive Peptide Database (MBPDB [16] and BIOPEP-UWM database of bioactive peptides (BIOPEP-UPW) [17]).

The potential of peptides to be bioactive was predicted using PeptideRanker software, and their theoretical bioactivity was expressed as score values calculated (from 0 to 1, with 1 being the most likely to be bioactive). Moreover, prediction of the toxicity was performed using ToxinPred.

### 2.3. Angiotensin Converting Enzyme (ACE)-Inhibitory Activity

The ACE-inhibitory activity of synthetic peptides was determined by the fluorescence protocol optimized by Sentandreu and Toldrá (2006) [18] and modified by Quirós et al. (2009) [19]. Briefly, the substrate Abz–Gly–Phe(NO_2_)–Pro was dissolved (0.45 mM) in 150 mM Tris and 1125 mM NaCl buffer (pH 8.3) and maintained at 4 °C until its use. ACE (1 U/mL) was diluted (0.04 U/mL) in 150 mM Tris buffer containing 0.1 μM ZnCl_2_ (pH 8.3). Forty microliters of sample (or MilliQ water for blank and control) were added to a black multi-well plate (Porvair, Leatherhead, UK). Then, 40 μL of ACE were added, and the reaction started after the addition of 160 μL of the substrate. The plate was incubated at 37 °C for 30 min, and the fluorescence was measured in a FLUOstar OPTIMA plate reader (BMG Labtechnologies GmbH, Offenburg, Germany) with 320 nm excitation and 420 nm emission filters. Data were processed with the FLUOstar Control version 1.32 R2 (BMG Labtech) software and expressed as IC_50_ (peptide concentration needed to inhibit 50% of the ACE activity).

### 2.4. In Vitro Antioxidant Activity

#### 2.4.1. Oxygen Radical Absorbance Capacity (ORAC)-FL Assay

An oxygen radical absorbance capacity (ORAC)-FL assay was used based on the protocol previously optimized [20]. The reaction was performed at 37 °C in 75 mM phosphate buffer (pH 7.4). The final assay mixture volume was 200 µL, containing 70 nM FL, 12 mM AAPH, and antioxidant [(Trolox, 1–8 µM) or sample (at different concentrations)]. Fluorescence was measured during 137 min in a FLUOstar OPTIMA plate reader (BMG Labtech) with 485 nm excitation and 520 nm emission filters. The equipment was controlled by the FLUOstar Control ver. 1.32 R2 software for fluorescence measurement. Samples were analyzed in triplicate. The final ORAC-FL value was expressed as μmol Trolox equivalents (TE) per μmol peptide.

#### 2.4.2. ABTS Assay

Antioxidant activity was measured using a previously optimized method [21] with some modifications. A mixture of 7 mM ABTS stock solution and 2.45 mM potassium persulfate was kept in the dark at room temperature for 12-16 h to form the ABTS radical cation (ABTS^•+^). The ABTS^•+^ solution was diluted in 5 mM of phosphate buffer solution (PBS) (pH 7.4) to an absorbance of 0.70 ± 0.02 at 734 nm at 30 °C. Two mL of diluted ABTS^•+^ solution were mixed with 20 μL of sample or Trolox (0–0.015 μmol), and the absorbance was recorded at 734 nm after 10 min incubation at 30 °C. The Trolox equivalent antioxidant capacity (TEAC) value was expressed as μmol TE per μmol peptide. Each sample was analyzed in triplicate.

### 2.5. Cell Culture

The human colorectal adenocarcinoma Caco-2 and the mouse macrophage RAW 264.7 cell lines were obtained from the American Type Culture Collection (ATCC, Rockville, MD, USA). Caco-2 and RAW264.7 cells were grown in High-Glucose DMEM supplemented with 10% (v:v) FBS, 1% (v:v) penicillin/streptomycin/amphotericin B, and 1% (v:v) non-essential amino acids solution. Cells were maintained in plastic 75-cm^2^ culture flasks at 37 °C in a humidified incubator containing 5% CO_2_ and 95% air.

### 2.6. Cell Treatment Conditions

Cells were incubated for 24 h with various concentrations of selected synthetic peptides. To evaluate both the direct and protective effects against oxidative stress, the incubation period was followed by 1.5 h-treatment with culture medium or *tert*-butyl hydroperoxide (*t*-BOOH, 1 mM for Caco-2 cells and 0.25 mM for RAW264.7 cells), respectively, and different biomarkers were evaluated.

#### 2.6.1. Cell Viability

Cell viability was determined using the MTT assay. Caco-2 and RAW264.7 cells were seeded onto 96-well plates (Corning Costar Corp., Corning, NY, USA) at a density of 1.0 × 10^4^ cells/cm^2^ and incubated for 9 days and 24 h, respectively. Then, cells were washed with PBS, treated with synthetic selected peptides (1–100 µM), and incubated for 24 h. Afterwards, culture medium was removed, and the cells were washed with PBS and incubated with medium (direct effects) or chemical oxidant (protective effects) at 37 °C for 1.5 h. At the end of the treatment time, 100 µL of MTT solution (0.5 mg/mL final concentration) were added to each well, and the plate was incubated for 2 h at 37 °C. The supernatant was aspirated, the formazan crystals were solubilized in DMSO:ethanol (1:1, v:v), and the absorbance was measured at 570 nm in a FLUOstar OPTIMA plate reader (BMG Labtech). Results were expressed as percentage of the control, considered as 100%. Samples were analyzed in triplicate.

#### 2.6.2. Determination of Intracellular Reactive Oxygen Species (ROS)

The intracellular ROS levels were detected using the ROS-sensitive fluorescent dye, DCFH, as previously described [22]. Caco-2 cells were plated in 48-well plates (density of 4.75 × 10^4^ cells/well) and RAW264.7 cells in 24-well plates (density of 2.0 × 10^5^ cells/well) and incubated for 7 days and 14 h, respectively. After this time, cells were treated with peptides as previously described for 24 h. Then, DCFH was dissolved in HBSS and added to cells at a final concentration of 5 µM solution. Cells were incubated for 30 min at 37 °C. The probe was removed, and cells were incubated with PBS (direct effects) or *t*-BOOH (protective effects) for 60 min, measuring the fluorescence after 60 min in a FLUOstar OPTIMA plate reader (BMG Labtech) with 485 nm excitation and 520 nm emission filters. The results were expressed as percentage of the control, which was considered as 100%. The assay was run in triplicate.

### 2.7. Statistical Analyses

All data were analyzed from three independent experiments. Results were expressed as the mean ± standard deviation (SD). Data were statistically analyzed by performing a one-way ANOVA test, followed by Tukey’s multiple comparison test with the IBM SPSS Statistics for Windows 23.0 (IBM Corporation, Armonk, NY, USA). A *p*-value of less than 0.05 was considered statistically significant. Significant differences of each concentration versus the control under the same experimental conditions were expressed by *** (*p* < 0.001), ** (*p* < 0.01), and * (*p* < 0.05).

## 3. Results and Discussion

### 3.1. In Silico Analysis of Synthetic Peptides

The physicochemical characteristics and predicted toxicity and biological activity of synthetic peptides are shown in Table 2. The bioactivity of peptides was predicted using the PeptideRanker program. There were differences in the theoretical bioactivity of peptides, with score values from 0.2453 to 0.9558. Eleven peptides, released from β-casein (β-CN), β-lactoglobulin (β-Lg), α-lactalbumin (α-La), and β-hemoglobin (β-Hg), were to be highly bioactive with a predicted bioactive score over 0.80. None of the 27 peptides were considered toxic, according to the software ToxinPred. The results of the in silico analysis by using the MBPDB and BIOPEP-UWM databases are shown in Table 3. Of the 27 synthetic peptides studied, 23 were already included into databases because of their opioid, ACE-inhibitory, antioxidant, anticancer, antidiabetic, or immunostimulating activities, among others. Some of them were found to exert two or more activities. Thus, α_s1_-CN-derived peptides RY, RYL, RYLG, YLGY and FYPEL exert both ACE-inhibitory and antioxidant activities [23,24,25,26,27]. ACE-inhibitory and opioid activities are exerted by sequences YGFLP [28] and YGFL [29,30]. Four of the analyzed peptides (RYLGY, LGY, YPFPGPI, and YLLF) have been reported to exert three or more activities. Among them, the multifunctional β-casein A2-derived peptide YPFPGPI is highlighted by exerting ACE and dipeptidyl peptidase IV (DPP-IV) inhibitory, anticancer, anxiolytic, immunomodulatory, opioid, antidiabetic, and satiating activities (Table 3). Only four peptides (YPFPGPIP, YPFVEP, YGFL, and YPW) were found to be novel, although they share active sequences with bioactive peptides released from the same source protein.

### 3.2. In Vitro ACE Inhibitory and Antioxidant Activities of Synthetic Peptides

The measured ACE-inhibitory and antioxidant activities of assayed peptides are shown in Table 4. Our study confirmed the ACE-inhibitory activity already reported for several of the analyzed fragments. Moreover, this effect was newly found in other sequences such as YLGYLE, YFYPE, YPFPGPIPN, YPFVEP, YGFL, and YLL. Potent activity (IC_50_ values lower than 10 µM) was determined for α_s1_-CN peptide YFYPEL (IC_50_ = 8.82 µM) and β-CN peptide YPFVEP (IC_50_ = 7.48 µM). These values were similar to those reported for well-known milk derived tripeptides VPP (IC_50_ = 9 µM) and IPP (IC_50_ = 5 µM) [59]. The presence of leucine, valine, and proline at the C-terminus could contribute to the high ACE-inhibitory activity shown by these two sequences, as ACE prefers substrates/competitive inhibitors with hydrophobic amino acids and/or proline at the three C-terminal positions [60]. Similarly, milk peptide HLPLP, containing leucine and proline at the C-terminus, showed an IC_50_ value of 41 µM [28]. As shown in Table 4, all peptides but sequence LLF presented antioxidant activity through a dual mechanism of action, hydrogen atom transfer (ORAC), and non-competitive electron transfer (ABTS). Our results confirmed the peroxyl radical scavenging activity already reported for eight of these peptides. Moreover, this property was newly found in 18 peptides whose ORAC values ranged from 1.09 ± 0.02 µmol TE/µmol peptide to 3.50 ± 0.02 µmol TE/µmol peptide. The highest value was determined for peptide YPW. The presence of tyrosine and tryptophan could determine its potent activity, as these two amino acids have been reported to be the main contributors to the peroxyl radical scavenging activity of food-derived peptides [20]. Moreover, the situation of these residues at the peptide chain could also influence the activity. Thus, when threonine was added to the C-terminus of peptide YPW, the ORAC value was reduced up to 3.19 ± 0.18 µmol TE/µmol peptide. This influence was also observed for peptides YLG and LGY. For peptide LG, the antioxidant behavior of the resultant peptides from the addition of tyrosine as a terminal residue was different. Thus, if this amino acid (Y) was added to the C-terminus, the ORAC value of peptide LGY was 2.00 ± 0.09 µmol TE/µmol peptide, while it was 0.93 ± 0.08 µmol TE/µmol peptide when tyrosine was added to the N-terminus (peptide YLG). When tyrosine was added at both termini, the ORAC value increased up to 2.96 ± 0.20 µmol TE/µmol peptide (Table 4). Our results confirmed the previously described importance of peptides parameters such as the their amino acid composition, sequence, and length in determining their antioxidative potential [61].

Unlike peroxyl radical scavening activity, none of the analyzed peptides had been previously reported to exert ABTS radical scavenging properties. TEAC values ranged from 0.73 ± 0.07 µmol TE/µmol peptide to 5.96 ± 0.35 µmol TE/µmol peptide (Table 4). As it has been described for the ORAC assay, the presence of tyrosine and tryptophane was responsible for the ABTS radical scavenging properties of analyzed peptides [21]. Among peptides whose antioxidant activity has been described for the first time in the present study, six sequences showed potent effects with ORAC and TEAC values higher than 2.0 µmol TE/µmol peptide. These sequences corresponded to α_s1_-CN peptides RYLGYLE, YLGYLE, and YFYPEL, β-CN peptide YGFLP, and β-Hg peptides YPW and YPWT. Five of these peptides were known by their opioid activity [31,32,36,37,42]. Additionally, anticancer and ACE-inhibitory activities have been reported for peptides RYLGYLE [33] and YGFLP [28], respectively. Thus, the antioxidant activity described in the present study would increase the functionality of these food-derived peptides. As a result of their multifunctionality allowing peptides to exert beneficial effects on different body systems, sequences RYLGYLE, YLGYLE, YFYPEL, and YPWT were selected to study in depth the mechanism of action involved in their antioxidant activity.

### 3.3. Antioxidant Activity of Synthetic Peptides in Cell Models

Two cell models, human colon adenocarcinoma Caco-2 and murine macrophages RAW264.7, were used to evaluate the protective effects of animal protein-derived peptides on the cell oxidative status under normal and chemical-induced conditions. The action of peptides on cell viability and ROS generation was studied. The direct effects of peptides RYLGYLE, YLGYLE, YFYPEL, and YPWT on Caco-2 and RAW264.7 cells viability were evaluated using the MTT assay. This assay provides a sensitive measurement of the metabolic status of the mitochondria, which reflects early cellular redox changes [62]. Treatment of Caco-2 and RAW264.7 cells with synthetic peptides did not evoke changes in cell viability, indicating that the concentrations selected (1–100 µM) did not damage cell integrity during the 24-h period of incubation.

**Figure 1:**
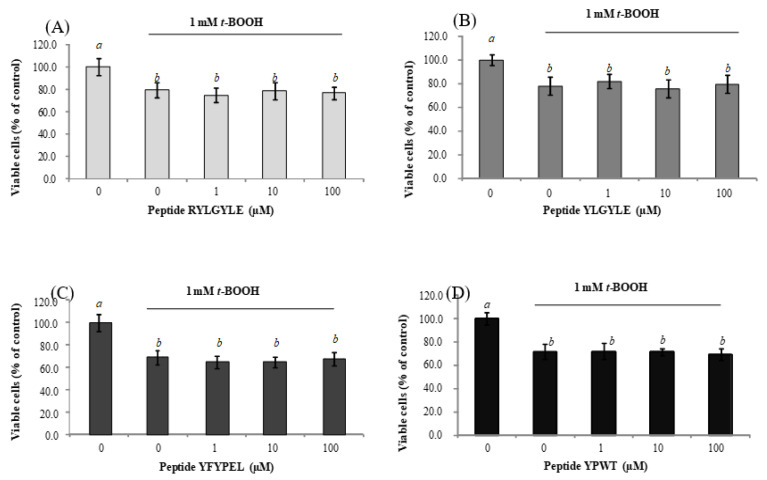
Dose-dependent effects of synthetic animal-protein derived peptides (**A**) RYLGYLE, (**B**) YLGYLE, (**C**) YFYPEL, and (**D**) YPWT on cell viability of stressed Caco-2 cells with 1 mM tert-butyl hydroperoxide (*t*-BOOH). Cells were pre-treated with peptides at concentrations ranged from 1 to 100 μM for 24 h. Results were expressed as the percentage of viable cells compared to control, which was considered as 100% (% control, mean ± standard deviation (SD), n = 3). Different letters indicate significant differences (*p* < 0.05; Tukey multiple comparison test).

To study the protective effects of peptides against chemical-induced oxidative damage in Caco-2 cells and macrophages, they were pre-incubated with peptides for 24 h, exposed to *t*-BOOH for 1.5 h, and then, cell viability was measured. As shown in Figure 1A–D, treatment of Caco-2 cells with *t*-BOOH (1 mM) provoked a significant reduction of cell viability of 25%, compared to non-stimulated cells.

**Figure 2:**
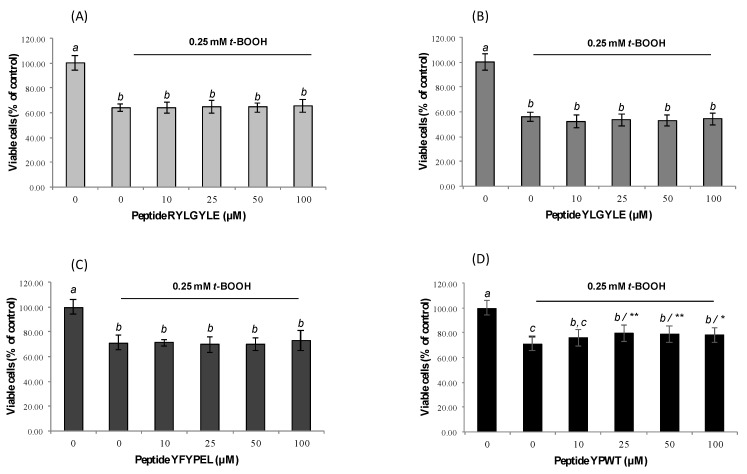
Dose-dependent effects of synthetic animal protein-derived peptides (**A**) RYLGYLE, (**B**) YLGYLE, (**C**) YFYPEL, and (**D**) YPWT on cell viability of stressed macrophages RAW264.7 with 0.25 mM tert-butyl hydroperoxide (*t*-BOOH). Cells were pre-treated with peptides at concentrations that ranged from 10 to 100 μM for 24 h. Results were expressed as the percentage of viable cells compared to control, considered as 100% (% control, mean ± standard deviation (SD), n = 3). Different letters indicate significant differences (*p* < 0.05) and ** (*p* < 0.01); * (*p* < 0.05) significant differences of each concentration versus control under the same experimental conditions (one-way ANOVA followed by Tukey’s multiple comparison test).

In a previous study in our lab, García-Nebot et al. (2014) [22] had reported a 20% reduction of the Caco-2 cells’ viability after 1.5-h treatment with 3 mM *t*-BOOH. A longer incubation time (6 h) with this chemical at concentrations of 0.1 and 4 mM has been also found to cause significant reductions of cell viability [63,64]. Similarly, the viability of macrophages was significantly reduced after treatment with *t*-BOOH (0.25 mM) by up to 44% (Figure 2A–D). Recent studies have also reported significant decreases (39%) of cell viability after treatment of RAW264.7 cells with 1 mM *t*-BOOH for 3 h [65]. Pre-treatment of Caco-2 cells with tested peptides before induction with *t*-BOOH for 1.5 h did not exert any protection from the effects of this chemical. In the case of macrophages, only treatment with peptide YPWT resulted in an increase of cell viability at concentrations between 25 and 100 µM. Thus, the percentage of viable cells increased from 70.97% (stimulated cells) to 79.58% (stimulated cells pre-treated with 25 µM YPWT) (Figure 2D). These results are in agreement with previous studies carried out with soybean protein-derived peptides that only found significant protection on *t*-BOOH-induced RAW264.7 cells at high doses [66].

**Figure 3:**
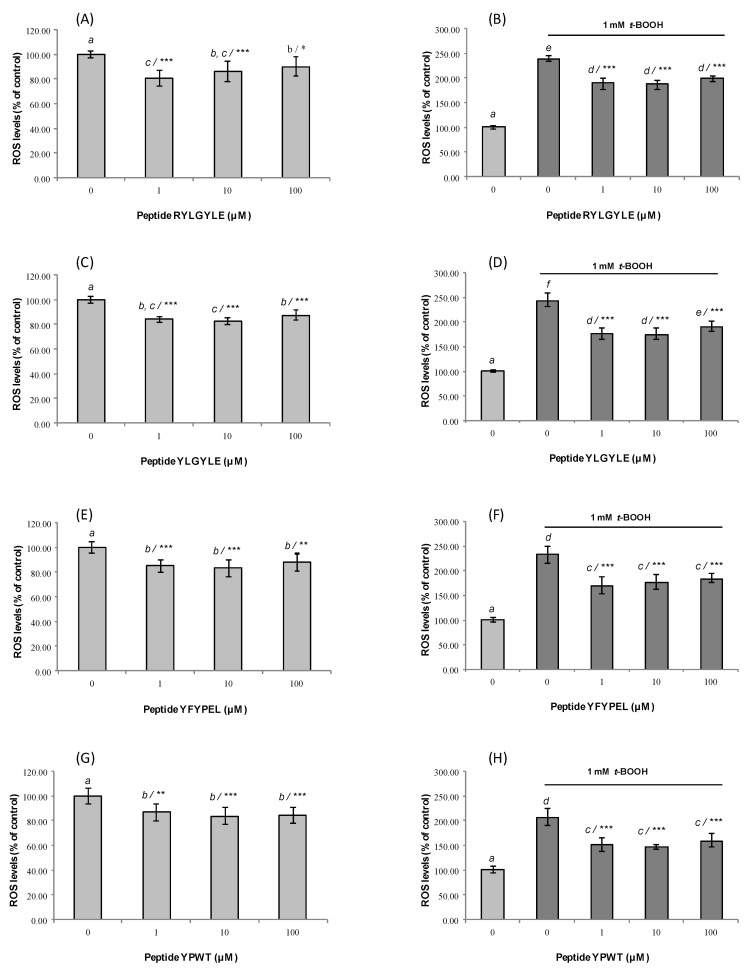
Dose-dependent effects of synthetic animal protein-derived peptides (**A**,**B**) RYLGYLE, (**C**,**D**) YLGYLE, (**E**,**F**) YFYPEL, and (**G**,**H**) YPWT on reactive oxygen species (ROS) production in non-stressed Caco-2 cells (**A**,**C**,**E**,**F**) and Caco-2 cells stressed with tert-butyl hydroperoxide (*t*-BOOH, 1 mM). Cells were pre-treated with peptides at concentrations that ranged from 1 to 100 μM for 24 h. Results were expressed as the percentage of ROS levels compared to control, which were considered as 100% (% control, mean ± standard deviation (SD), n = 3. Different letters indicate significant differences (*p* < 0.05) and *** (*p* < 0.001); ** (*p* < 0.01); * (*p* < 0.05) indicate significant differences of each concentration versus control under the same experimental conditions (one-way ANOVA followed by Tukey’s multiple comparison test).

In order to understand the potential mechanism of cytoprotective action exerted by peptides, the intracellular ROS generation was evaluated in normal cells and cells exposed to *t*-BOOH after pre-treatment with synthetic peptides for 24 h. The direct evaluation of intracellular ROS is recognized as a good indicator of the oxidative damage to living cells [67]. In our study, measurement of the intracellular ROS levels was carried out using DCFH as a fluorescent probe that once added to intact cells, crosses cell membranes and is oxidized to highly fluorescent dichlorofluorescein (DCF) in the presence of ROS [68]. In Caco-2 cells under normal conditions, four peptides caused a significant reduction of ROS levels at all concentrations used (Figure 3A,C,E,G).

**Figure 4:**
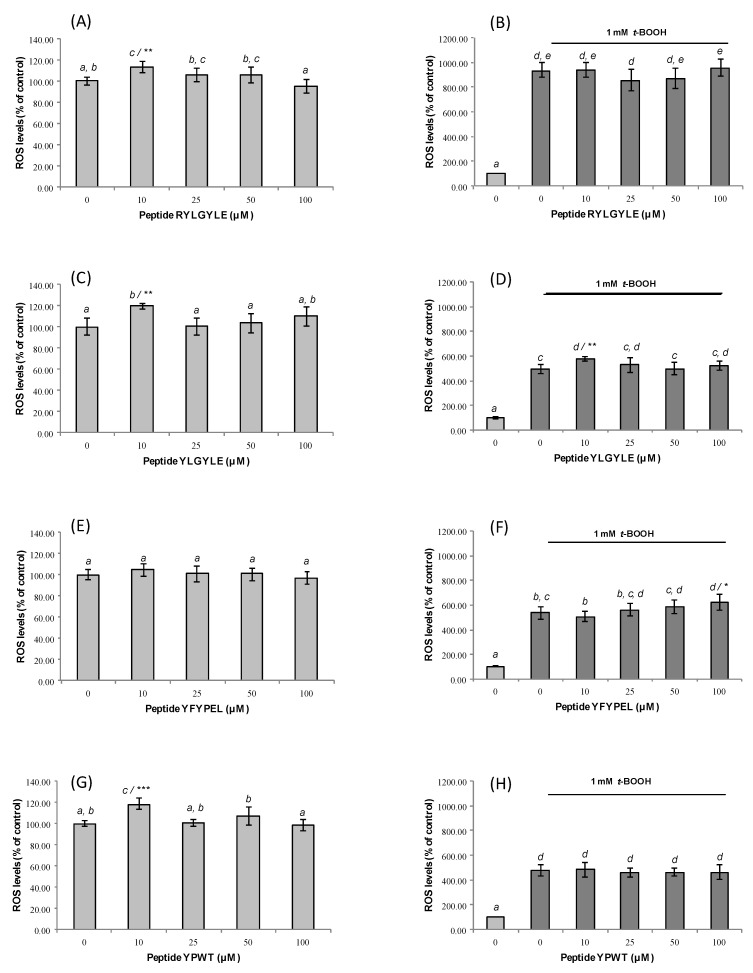
Dose-dependent effects of synthetic animal-protein derived peptides (**A**,**B**) RYLGYLE, (**C**,**D**) YLGYLE, (**E**,**F**) YFYPEL, and (**G**,**H**) YPWT on ROS production in non-stressed macrophages RAW264.7 (**A**,**C**,**E**,**F**) and macrophages RAW264.7 stressed with tert-butyl hydroperoxide (*t*-BOOH, 0.25 mM). Cells were pre-treated with peptides at concentrations that ranged from 10 to 100 μM for 24 h. Results were expressed as percentage of ROS levels compared to control, which was considered as 100% (% control, mean ± standard deviation (SD), n = 3. Different letters indicate significant differences (*p* < 0.05) and *** (*p* < 0.001); ** (*p* < 0.01); * (*p* < 0.05) significant differences of each concentration versus control under the same experimental conditions (one-way ANOVA followed by Tukey’s multiple comparison test).

In the case of fragments RYLGYLE and YLGYLE, the highest reduction (19.3% and 17.3%) was observed after treating cells with low concentrations of these peptides (1 and 10 µM, respectively), compared to non-treated cells. However, these two peptides at 10 µM provoked an increase of ROS levels in non-stressed RAW264.7 (Figure 4A,C) cells. These results indicated that in addition to the peptide dose, the type of cell line could influence the antioxidant activity of the peptides.

The chemical exposition of Caco-2 cells to t-BOOH for 1.5 h significantly increases ROS levels (untreated Caco-2 cells 100.00 ± 4.15%; treated Caco-2 cells with 1 mM t-BOOH 230.19 ± 13.61%) (*p* < 0.05) (Figure 3B,D,F,H). The pre-treatment with animal protein-derived peptides at all assayed doses for 24 h significantly neutralized the ROS-generating ability of the chemical, but no dose-dependence was observed. The highest reduction (≈68.5%) was observed for cells treated with peptide YLGYLE at 1 and 10 µM, which had ROS levels of 175.06 ± 11.13% and 174.44 ± 11.60%, respectively, compared to non-peptide-treated cells (Figure 3D). In the case of macrophages, the exposition to *t*-BOOH resulted in a higher increase of ROS levels (untreated RAW264.7 cells 100.00 ± 4.77%; treated RAW264.7 cells with 0.25 mM *t*-BOOH 501.27 ± 44.20%) (Figure 4B,D,F,H). This result was similar to that found previously by Indiano-Romacho et al. (2019) [66]. Tested peptides did not show any antioxidant activity in macrophages. No significant ROS level decreases versus control were observed after treatment with peptides both in untreated and treated RAW264.7 cells with *t*-BOOH. This tendency is in disagreement with that observed both in the Caco-2 cell model and in the in vitro antioxidant activity measured by ORAC-FL and ABTS assays; besides that, cell viability is not compromised because of the MTT results. Among cases that can help appreciate these differences, fragment YFYPEL shows one of the most remarkable activities in the stressed Caco-2 cell model, significantly lowering ROS levels (from 231.58 ± 18.40% up to 168.88 ± 17.80% at 1 µM) and showing significant in vitro antioxidant activity (2.66 ± 0.16 and 2.59 ± 0.17 µmol TE/µmol peptide for ORAC and TEAC, respectively), but without any antioxidant activity in homologous conditions in a RAW264.7 cell model. It would be interesting to evaluate the suitability of this cell model for the study of antioxidant capacity.

## 4. Conclusions

In summary, our results have demonstrated the multifunctionality of different bioactive peptides by an approach combining in silico and in vitro assays. By the in silico analysis, different activities such as ACE inhibitory, antioxidant, opioid, and anticancer, among others, were found to be exerted by most of analyzed peptides.

Moreover, four novel peptides (YPFPGPIP, YPFVEP, YGFL, and YPW) had not been previously defined as bioactive peptides. The ACE-inhibitory and the antioxidant activities of peptides mediated through a dual mechanism of action were confirmed by in vitro assays. Four of these peptides, RYLGYLE, YLGYLE, YFYPEL, and YPWT, were selected by their potent activity, which was confirmed in the gut epithelial cell model Caco-2, protecting cells from the oxidative damage caused by chemical agents. Future animal models would be required to confirm the multifunctionality of food-derived peptides and their protective capacity against oxidative stress-associated diseases.

## Figures and Tables

**Table 1:** Source protein, fragment, sequence, molecular mass, and purity of synthetic peptides used in the present study.

Source Protein	Sequence	Fragment	Molecular Mass (Da)	Purity (%)
α_S1_-casein	RY	f(90–91)	337.39	99.1
	RYL	f(90–92)	450.57	99.6
	RYLG	f(90–93)	507.64	99.4
	RYLGY	f(90–94)	670.83	88.0
	RYLGYLE	f(90–96)	913.14	73.4
	YLG	f(91–93)	351.44	92.0
	YLGY	f(91–94)	514.63	97.8
	YLGYLE	f(91–96)	756.94	98.9
	LGY	f(92–94)	351.44	98.9
α_S1_-casein	AYFYPE	f(143–148)	788.92	99.2
	YFYPEL	f(144–149)	831.01	100.0
	FYPEL	f(145–149)	667.82	99.0
β-casein A2	YPFPGPI	f(60–66)	790.02	96.0
	YPFPGPIP	f(60–67)	887.15	92.9
	YPFPGPIN	f(60–68)	904.14	94.0
β-casein	YPFVE	f(51–55)	653.79	95.0
	YPFVEP	f(51–56)	750.92	100.0
	YGFL	f(59–62)	498.63	98.5
	YGFLP	f(59–63)	595.76	100.0
	YPVEPF	f(114–119)	750.92	92.3
α-La	YGLF	f(50–53)	498.63	97.6
β-Lg	YLL	f(102–104)	407.55	100.0
	YLLF	f(102–105)	554.74	100.0
	LLF	f(103–105)	391.55	96.5
β-Hg	YPW	f(34–36)	464.55	84.5
	YPWT	f(34–37)	565.67	91.7
	PWT	f(35–37)	402.48	86.3

**Table 2:** Physicochemical characteristics and predicted biological activity and toxicity of synthetic peptides derived from food sources (PeptideRanker and ToxinPred databases).

Peptide	Hydrophobicity	Hydrophilicity	Charge	pI ^1^	Toxicity Prediction	Activity Prediction
RY	−0.87	0.35	1.00	9.10	Non toxin	0.5437
RYL	−0.40	−0.37	1.00	9.10	Non toxin	0.5627
RYLG	−0.26	−0.27	1.00	9.10	Non toxin	0.5215
RYLGY	−0.21	−0.68	1.00	8.93	Non toxin	0.4505
RYLGYLE	−0.16	−0.31	0.00	6.35	Non toxin	0.2453
YLG	0.24	−1.37	0.00	5.88	Non toxin	0.6404
YLGY	0.18	−1.60	0.00	5.87	Non toxin	0.6138
YLGYLE	0.11	−0.87	−1.00	4.00	Non toxin	0.3219
LGY	0.24	−1.37	0.00	5.88	Non toxin	0.5959
AYFYPE	0.04	−0.77	−1.00	4.00	Non toxin	0.7126
YFYPEL	0.08	−0.98	−1.00	4.00	Non toxin	0.7603
FYPEL	0.09	−0.72	−1.00	4.00	Non toxin	0.7939
YPFPGPI	0.19	−0.94	0.00	5.88	Non toxin	0.9175
YPFPGPIP	0.15	−0.82	0.00	5.88	Non toxin	0.8990
YPFPGPIPN	0.08	−0.80	0.00	5.88	Non toxin	0.8061
YPFVE	0.10	−0.66	−1.00	4.00	Non toxin	0.4339
YPFVEP	0.07	−0.55	−1.00	4.00	Non toxin	0.5114
YGFL	0.33	−1.65	0.00	5.88	Non toxin	0.9558
YGFLP	0.25	−1.32	0.00	5.88	Non toxin	0.9432
YPVEPF	0.07	−0.55	−1.00	4.00	Non toxin	0.6345
YGLF	0.33	−1.65	0.00	5.88	Non toxin	0.9537
YLL	0.36	−1.97	0.00	5.88	Non toxin	0.6000
YLLF	0.42	−2.10	0.00	5.88	Non toxin	0.9038
LLF	0.56	−2.03	0.00	5.88	Non toxin	0.9389
YPW	0.11	−1.90	0.00	5.88	Non toxin	0.9751
YPWT	0.04	−1.52	0.00	5.88	Non toxin	0.8795
PWT	0.04	−1.27	0.00	5.88	Non toxin	0.8928

^1^ pI: Isoelectric point.

**Table 3:** Predicted biological activity of synthetic peptides derived from food sources using Milk Bioactive Peptide Database (MBPDB) and BIOPEP-UWM database of bioactive peptides.

Sequence	Biological Activity	Results	Reference
RY	ACE inhibitory	IC_50_^a^ = 51.00 µM */54.43 µM **	[23] */[24] **
	Antioxidant	ORAC = 1.94 µmol TE/µmol peptide **	[24] **
RYL	ACE inhibitory	IC_50_^a^ = 3.31 µM */106.64 µM **	[25] */[26] **
	Antioxidant	ORAC = 1.75 µmol TE/µmol peptide **	[24] **
RYLG	ACE inhibitory	IC_50_^a^ = 224.69 µM **	[24] **
	Antioxidant	ORAC = 1.67 µmol TE/µmol peptide **	[24] **
RYLGY	ACE inhibitory	IC_50_^a^ = 0.71 µM *^,^**	[26] *^,^**
	Antioxidant	ORAC = 2.83 µmol TE/µmol peptide **	[24] **
	Opioid	Stimulation of mucin secretion **	[31] **
RYLGYLE	Opioid	IC_50_^b^ = 1.2 µM *	[32] *
	Anticancer	Decrease of breast cancer cell proliferation **	[33] **
YLG	Antioxidant	ORAC = 1.38 µmol TE/µmol peptide **	[24] **
YLGY	ACE inhibitory	IC_50_^a^ = 41.86 µM *^,^**	[24] *^,^**
	Antioxidant	ORAC = 1.46 µmol TE/µmol peptide **	[26] **
YLGYLE	Opioid	IC_50_^b^ = 45.00 µM *	[32] *
		Stimulation of mucin secretion **	[31] **
LGY	Immunostimulating	n.d.	[34] *
	ACE inhibitory	IC_50_^a^ = 21.46 µM **	[24] **
	Antioxidant	ORAC = 2.31 µmol TE/µmol peptide **	[24] **
AYFYPE	ACE inhibitory	IC_50_^a^ = 106.00 µM *^,^**/260.82 µM **	[35] *^,^**/[24] **
YFYPEL	Antioxidant	DPPH value = 79.20 µM **	[27] **
	Opioid	Increase MUC5AC expression	[36,37] **
FYPEL	ACE inhibitory	IC_50_^a^ = 80.60 µM **	[24] **
	Antioxidant	ORAC = 1.77 µmol TE/µmol peptide **/DPPH = 127.50 µM **	[24] **/[27] **
YPFPGPI	ACE inhibitory	IC_50_^a^ = 500.00 µM **	[38] **
	Anticancer	Decrease of breast cancer cell proliferation **	[33] **
	Anxiolytic	Induction of inflammatory immune response in gut **	[39] **
	Immunomodulatory	Inhibition/stimulation of lymphocyte proliferation at low/high concentrations **	[40] **
	Opioid	Stimulation of lymphocyte proliferation ^d^ = −21/+26 **	[41] **
		IC_50_^c^ = 14 µM **	[42] **
		Increase of jejunal mucus secretion and mucus discharge **	[43] **
		Increase of MUC2 and MUC3 expression in DHE cells **	
		Increase of MUC5A expression in HT29-MTX cells **	[44] **
		Stimulation of mucin secretion **	[36,45] **
	Antidiabetic	Reduction of pancreas MDA level in diabetic rats **	[46] **
	Satiating	Induction of CCK-8 **	[47] **
YPFPGPIP	n.d.	n.d	n.d
YPFPGPIPN	ACE inhibitory	IC_50_^a^ = 14.80 µM **	[48] **
	Antidiabetic	IC_50_^e^ = 6.70 µM **	[49] **
YPFVE	Opioid	Stimulation of mucin secretion **	[50] **
YPFVEP	n.d.	n.d	n.d
YGFL	n.d.	n.d	n.d
YGFLP	ACE inhibitory	IC_50_^a^ = 260.00 µM *	[28] *
	Opioid agonist	n.d.	n.d.
YPVEPF	Antidiabetic	IC_50_^e^ = 124.70 µM *	[51] *
	Opioid	IC_50_^c^ = 59.00 µM **	[52] **
		Increase of MUC4 expression **	[53] **
YGLF	ACE inhibitory	IC_50_^a^ = 733.30 µM *	[30] *
	Opioid agonist	IC_50_^c^ = 300.00 µM **	[29] **
YLL	Antioxidant	FRAP = 81.76 mmol Fe/mol peptide **	[54] **
YLLF	ACE inhibitory	IC_50_^a^ = 171.80 µM *	[30] *
	Opioid agonist	IC_50_^c^ = 160.00 µM *	[29] *
		Stimulation of mucin secretion **	[36,50,55] **
	Cytotoxic	Stimulation of murine splenocytes **	[56] **
LLF	ACE inhibitory	IC_50_^a^ = 79.80 µM *	[57] *
YPW	n.d.	n.d.	n.d.
YPWT	Opioid	IC_50_^c^ = 45.20 µM *	[58] *
PWT	Antioxidant	Inhibition of linoleic acid peroxidation *	[48] *

* According to BIOPEP-UWM database; ** According to Milk Bioactive Peptide Database (MBPDB); IC_50_^a^: Values (µM) are given for peptide concentrations inhibiting the angiotensin-converting enzyme (ACE) activity by 50%; IC_50_^b^: Values (µM) is given for peptide concentration inhibiting (3H)-dihydromorphine binding, instead of (3H)-naloxone, by 50%; IC_50_^c^: Values (µM) are given for peptide concentrations inhibiting (3H)-naloxone binding by 50%; Stimulated lymphocyte proliferation ^d^: % stimulation (+) and inhibition (–), respectively, compared to control; IC_50_^e^: Values (µM) are given for peptide concentration required to inhibit 50% of dipeptidyl peptidase IV (DPP-IV); n.d. No available data. ORAC: oxygen radical absorbance capacity.

**Table 4:** ACE-inhibitory activity (expressed as µM) and antioxidant activity (expressed as µmol Trolox equivalents (TE)/µmol peptide of synthetic animal-protein derived peptides.

Sequence	ACE ^1^ Inhibitory Activity (IC_50_-µM)	Antioxidant Activity (µmol TE^2^/µmol Peptide)	
		ORAC	TEAC
RY	*	1.83 ± 0.13	1.38 ± 0.03
RYL	*	1.72 ± 0.14	1.90 ± 0.03
RYLG	*	1.70 ± 0.11	2.91 ± 0.21
RYLGY	3.08 ± 0.11	2.97 ± 0.09	1.38 ± 0.14
RYLGYLE	*	2.88 ± 0.07	3.10 ± 0.01
YLG	*	0.93 ± 0.08	1.40 ± 0.04
YLGY	9.87 ± 0.31	2.96 ± 0.20	2.14 ± 0.11
YLGYLE	85.76 ± 4.66	2.28 ± 0.22	5.96 ± 0.35
LGY	26.10 ± 0.83	2.00 ± 0.09	1.54 ± 0.01
AYFYPE	774.36 ± 38.22	2.60 ± 0.13	1.99 ± 0.19
YFYPEL	8.82 ± 0.58	2.66 ± 0.16	2.59 ± 0.17
FYPEL	62.00 ± 6.27	1.88 ± 0.13	1.74 ± 0.04
YPFPGPI	685.91 ± 102.91	1.91 ± 0.14	1.62 ± 0.08
YPFPGPIP	224.05 ± 43.94	1.09 ± 0.02	1.86 ± 0.01
YPFPGPIN	378.65 ± 11.15	1.26 ± 0.06	1.22 ± 0.17
YPFVE	*	1.53 ± 0.15	1.78 ± 0.02
YPFVEP	7.48 ± 0.03	1.96 ± 0.14	1.43 ± 0.08
YGFL	292.53 ± 0.83	1.42 ± 0.04	2.12 ± 0.03
YGFLP	272.39 ± 0.25	2.27 ± 0.15	2.22 ± 0.02
YPVEPF	*	1.62 ± 0.09	1.75 ± 0.10
YGLF	*	0.89 ± 0.01	2.08 ± 0.06
YLL	518.54 ± 3.50	0.78 ± 0.03	2.55 ± 0.29
YLLF	n.d.	0.91 ± 0.04	1.96 ± 0.30
LLF	94.79 ± 2.97	**	**
YPW	*	3.50 ± 0.02	2.32 ± 0.09
YPWT	*	3.19 ± 0.18	3.89 ± 0.10
PWT	*	2.15 ± 0.07	0.73 ± 0.07

^1^ ACE: Angiotensin-converting enzyme; ^2^ TE: Trolox equivalents; TEAC: Trolox equivalent antioxidant capacity; n.d. Activity not determined. * ACE-inhibitory activity not detected at the highest peptide concentration analyzed (1000 µM). ** Antioxidant activity not detected at the highest peptide concentration analyzed (0.20 µmol).

## Supplementary Materials

The following are available online at https://www.mdpi.com/2304-8158/9/8/991/s1, Figure S1: RP-HPLC-MS/MS analysis of synthesized peptide YPFVE at concentration of 0.2 mg/mL (A) UV chromatogram obtained at wavelength of 214 nm; (B) Peak integration of UV chromatogram; (C) Total ion current chromatogram; (D) Average mass spectrum; (E) MS/MS spectrum of selected ion m/z 654.3 (retention time 38.3 min); (F) MS/MS fragment ions identification in sequence YPFVE.
