# Supplementary material for: In Silico and In Vitro Analysis of Multifunctionality of Animal Food-Derived Peptides
Source: Foods. 2020 Jul 24;9(8):991. doi: 10.3390/foods9080991 (PMC7466261; doi:10.3390/foods9080991)
Supplement: Supplementary file 1 [file foods-09-00991-s001.pdf]

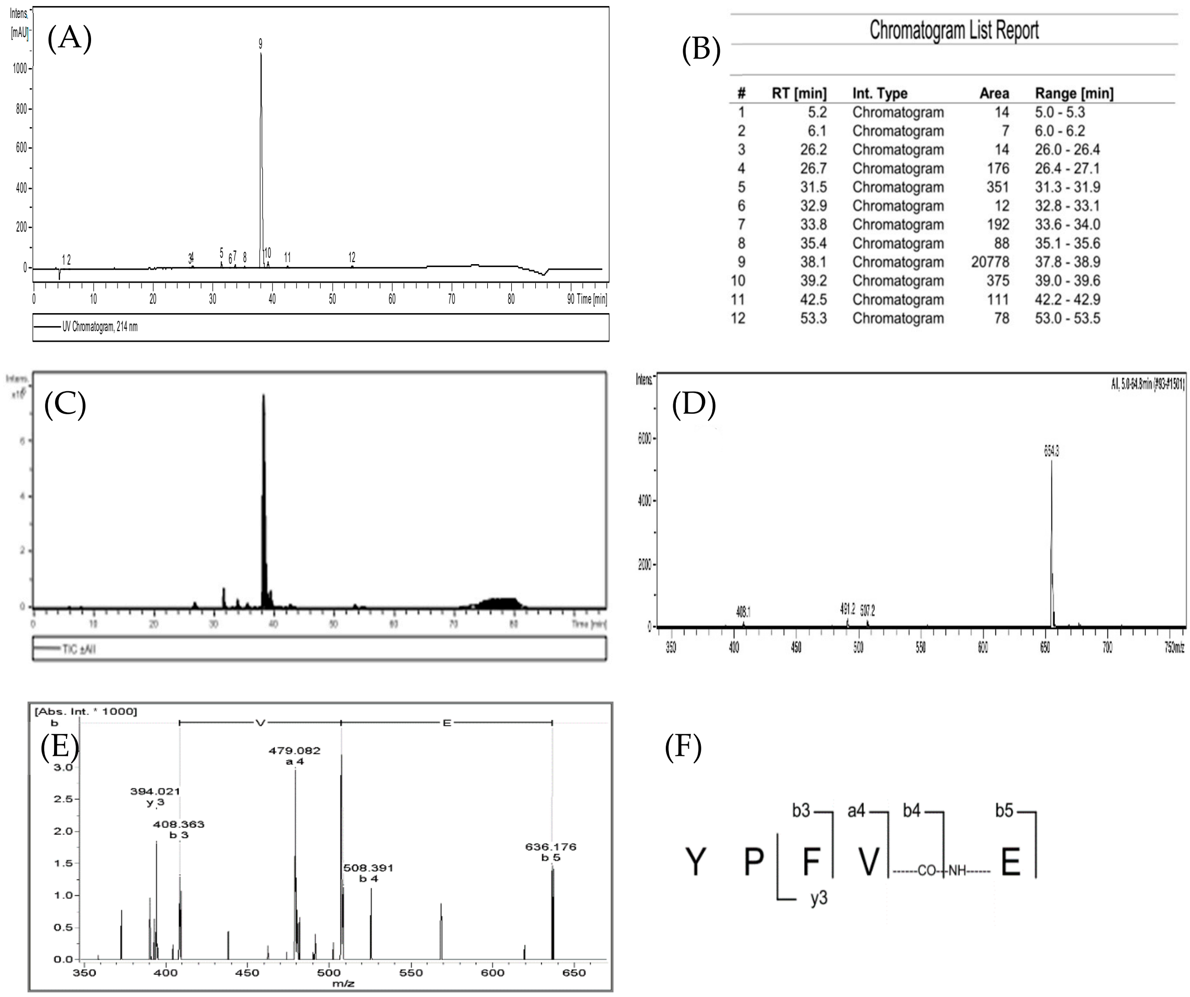


Chromatogram List Report

| # | RT [min] | Int. Type | Area | Range [min] |
|---|---|---|---|---|
| 1 | 5.2 | Chromatogram | 14 | 5.0 - 5.3 |
| 2 | 6.1 | Chromatogram | 7 | 6.0 - 6.2 |
| 3 | 26.2 | Chromatogram | 14 | 26.0 - 26.4 |
| 4 | 26.7 | Chromatogram | 176 | 26.4 - 27.1 |
| 5 | 31.5 | Chromatogram | 351 | 31.3 - 31.9 |
| 6 | 32.9 | Chromatogram | 12 | 32.8 - 33.1 |
| 7 | 33.8 | Chromatogram | 192 | 33.6 - 34.0 |
| 8 | 35.4 | Chromatogram | 88 | 35.1 - 35.6 |
| 9 | 38.1 | Chromatogram | 20778 | 37.8 - 38.9 |
| 10 | 39.2 | Chromatogram | 375 | 39.0 - 39.6 |
| 11 | 42.5 | Chromatogram | 111 | 42.2 - 42.9 |
| 12 | 53.3 | Chromatogram | 78 | 53.0 - 53.5 |

Figure S1. RP-HPLC-MS/MS analysis of synthesized peptide YPFVE at concentration of 0.2 mg/mL (A) UV chromatogram obtained at wavelength of 214 nm; (B) Peak integration of UV chromatogram; (C) Total ion current chromatogram; (D) Average mass spectrum; (E) MS/MS spectrum of selected ion m/z 654.3 (retention time 38.3 minutes); (F) MS/MS fragment ions identification in sequence YPFVE.
